# Supplementary material for: EnzML: multi-label prediction of enzyme classes using InterPro signatures
Source: BMC Bioinformatics. 2012 Apr 25;13:61. doi: 10.1186/1471-2105-13-61 (PMC3483700; doi:10.1186/1471-2105-13-61)
Supplement: Addtional file 5 — The Java code to format the data files, evaluate and predict. The file enzml_java_code.tar.gz contains the Java code used to format database data to ARFF and XML formats, to execute cross and train-test (jackknife) evaluations and to record evaluation results to database. More information is included in the readme.txt file and the Javadoc files. The code can be used with a MySQL database. To use a different database software, other JDBC drivers might be required. [file 1471-2105-13-61-S5.gz › java_code/enzml2011/doc/uk/ac/ed/inf/csb/taweka/TaWeka.html]

TaWeka


---


|  |  |  |
| --- | --- | --- |
| **Package** \| **Class** \| **Use** \| **Tree** \| **Deprecated** \| **Index** \| **Help** | |  |
| **PREV CLASS**   **NEXT CLASS** | **FRAMES**    **NO FRAMES**     **All Classes** |  |
| SUMMARY: NESTED \| FIELD \| CONSTR \| METHOD | DETAIL: FIELD \| CONSTR \| METHOD |  |


---


## uk.ac.ed.inf.csb.taweka Class TaWeka

```
java.lang.Object
  uk.ac.ed.inf.csb.taweka.TaWeka
```

---

``` public class TaWeka extends java.lang.Object ```

General information about TaWeka (author, documentation web page, version etc)

**Version:**
:   22 Apr 2010

**Author:**
:   Luna De Ferrari luna.deferrari-at-ed.ac.uk

---

| **Field Summary** | |
| --- | --- |
| `static java.lang.String` | `AUTHOR` |
| `static java.lang.String` | `TAWEKA_NAME` |
| `static java.lang.String` | `VERSION` |
| `static java.lang.String` | `WEB_PAGE` |


**Constructor Summary**

`TaWeka()`


**Method Summary**

**Methods inherited from class java.lang.Object**

`equals, getClass, hashCode, notify, notifyAll, toString, wait, wait, wait`

**Field Detail**

### TAWEKA\_NAME

```
public static final java.lang.String TAWEKA_NAME
```

**See Also:**: Constant Field Values

---


### AUTHOR

```
public static final java.lang.String AUTHOR
```

**See Also:**: Constant Field Values

---


### WEB\_PAGE

```
public static final java.lang.String WEB_PAGE
```

**See Also:**: Constant Field Values

---


### VERSION

```
public static final java.lang.String VERSION
```

**See Also:**: Constant Field Values


**Constructor Detail**

### TaWeka

```
public TaWeka()
```


---


|  |  |  |
| --- | --- | --- |
| **Package** \| **Class** \| **Use** \| **Tree** \| **Deprecated** \| **Index** \| **Help** | |  |
| **PREV CLASS**   **NEXT CLASS** | **FRAMES**    **NO FRAMES**     **All Classes** |  |
| SUMMARY: NESTED \| FIELD \| CONSTR \| METHOD | DETAIL: FIELD \| CONSTR \| METHOD |  |


---
